# The experiences of women with maternal near miss and their perception of quality of care in Kelantan, Malaysia: a qualitative study

**DOI:** 10.1186/s12884-017-1377-6

**Published:** 2017-06-15

**Authors:** Mohd Noor Norhayati, Nik Hussain Nik Hazlina, Ab Razak Asrenee, Zaharah Sulaiman

**Affiliations:** 1 Department of Family Medicine, School of Medical Sciences, Health Campus, Universiti Sains Malaysia, 16150 Kubang Kerian, Kelantan Malaysia; 2 Women’s Health Development Unit, School of Medical Sciences, Universiti Sains Malaysia, Health Campus, 16150 Kubang Kerian, Kelantan Malaysia; 3 Department of Psychiatry, School of Medical Sciences, Health Campus, Universiti Sains Malaysia, 16150 Kubang Kerian, Kelantan Malaysia

**Keywords:** Maternal near miss, Experience, Quality of care, Social support, Phenomenological approach

## Abstract

**Background:**

Maternal mortality has been the main way of ascertaining the outcome of maternal and obstetric care. However, maternal morbidities occur more frequently than maternal deaths; therefore, maternal near miss was suggested as a more useful indicator for the evaluation and improvement of maternal health services. Our study aimed to explore the experiences of women with maternal near miss and their perception of the quality of care in Kelantan, Malaysia.

**Methods:**

A qualitative phenomenological approach with in-depth interview method was conducted in two tertiary hospitals in Kelantan, Malaysia. All women admitted to labour room, obstetrics and gynaecology wards and intensive care units in 2014 were screened for the presence of any vital organ dysfunction or failure based on the World Health Organization criteria for maternal near miss. Pregnancy irrespective of the gestational age was included. Women younger than 18 years old, with psychiatric disorder and beyond 42 days of childbirth were excluded.

**Results:**

Thirty women who had experienced maternal near miss events were included in the analysis. All were Malays between the ages of 22 and 45. Almost all women (93.3%) had secondary and tertiary education and 63.3% were employed. The women’s perceptions of the quality of their care were influenced by the competency and promptness in the provision of care, interpersonal communication, information-sharing and the quality of physical resources. The predisposition to seek healthcare was influenced by costs, self-attitude and beliefs.

**Conclusions:**

Self-appraisal of maternal near miss, their perception of the quality of care, their predisposition to seek healthcare and the social support received were the four major themes that emerged from the experiences and perceptions of women with maternal near miss. The women with maternal near miss viewed their experiences as frightening and that they experienced other negative emotions and a sense of imminent death. The factors influencing women’s perceptions of quality of care should be of concern to those seeking to improve services at healthcare facilities. The addition of a maternal near miss case review programme, allows for understanding on the factors related to providing care or to the predisposition to seek care; if addressed, may improve future healthcare and patient outcomes.

**Electronic supplementary material:**

The online version of this article (doi:10.1186/s12884-017-1377-6) contains supplementary material, which is available to authorized users.

## Plain English summary

This study explores the experiences and health care received by women who nearly died as a result of complications during pregnancy and childbirth. It was conducted in two general hospitals in Kelantan, Malaysia during 2014. The narratives were obtained from 30 women between the ages of 22 and 45. They viewed their experiences as frightening along with a sense of imminent death. The views regarding health care were affected by the promptness in delivering care, communication between the patients and hospital staffs, the information provided and condition of physical resources. Support from husband and families reduce the women’s suffering. The tendency to seek care was affected by the cost of health care, attitude and beliefs. Religious faith plays an important role in these situations.

## Background

In the past two decades, maternal near miss has gained interest in the area of maternal health. Notably, the decrease in maternal deaths and the extremely low maternal mortality ratio in developed countries have stimulated interest in investigating cases of maternal near misses [1].

Since maternal morbidities occur more frequently than maternal deaths, maternal near miss was suggested as a more useful indicator for the evaluation and improvement of maternal health services than the maternal death [1]. Secondly, investigating the maternal care would be less threatening to health providers because the woman survived [2]. A maternal near miss is defined as ‘a woman who nearly died but survived a complication that occurred during pregnancy, childbirth or within 42 days after termination of pregnancy’ [3]. The World Health Organization (WHO) criteria for identifying maternal near miss cases are based dysfunction or failure of any vital organ (circulatory, respiratory, cardiac, renal, hepatic, central nervous, metabolic and haematological dysfunctions) and the resulting complications. A block of clinical, laboratory and management-related markers was developed to enable the identification of severe cases [3].

The WHO near miss indicators provide an estimate on the quality of maternal care [4]. In Malaysia, the maternal near miss mortality ratio was 23.5 and maternal mortality index was 4.1%. The low index (< 5%) indicates better quality of care with fewer women with severe conditions dying [4]. Quality of care during childbirth in health facilities is reflected in the availability of physical infrastructure, supplies, management, human resources along with the knowledge, skills and capacity to deal with pregnancy and childbirth [5]. It is estimated that one third of maternal deaths are considered potentially preventable [6].

A meta-synthesis of nine qualitative studies published from 1980 until 2013 on the experiences of women with severe maternal morbidity has reported poor mental and physical health outcomes [7]. The review refers severe maternal morbidity to broad category of women who suffered complications related to pregnancy, delivery and puerperium. The occurrence of disagreeable physical experiences among the women that was triggered by a severe pregnancy complication has leads to the feeling of impending death and fear. The loss of a baby has negatively influenced the women’s perspectives and their coping mechanisms. Some women turned to religious beliefs in overcoming their difficulties [7].

The current study aimed to explore the experiences of women with maternal near miss events and their perceptions of the quality of care in Kelantan, Malaysia. We applied the standard definition and identification criteria of maternal near miss based on the WHO that is aligned with the ‘maternal death’ definition. This study was the first qualitative study at the national level; thus, providing a paramount contribution to the current knowledge on maternal health. The findings allow for better understanding in the experiences of women with maternal near miss, strengths and weaknesses in the healthcare system.

## Methods

### Setting and participants

A qualitative phenomenological approach with in-depth interview method was conducted in two tertiary hospitals, Raja Perempuan Zainab II Hospital and Universiti Sains Malaysia Hospital, Kelantan, Malaysia. All women admitted to labour room, obstetrics and gynaecology wards and intensive care units in the healthcare facilities between January and December 2014 were screened for the presence of any vital organ dysfunction or failure based on the WHO criteria for maternal near miss. Women with maternal near miss aged more than 18, irrespective of period of gestation were included. Those with history of diagnosed psychiatric disorder and beyond 42 days after termination of pregnancy were excluded. Purposive sampling was applied.

### Data collection

During hospitalization, prior to discharge, women with maternal near miss were identified based on the study’s eligibility criteria. The interviewer reviewed the home- and hospital-based medical records prior to the interview to have an idea on the medical problems and to ensure that the women were medically stable. We used a standard semi-structured interview guide covering topics on antenatal care experiences in primary health facilities, childbirth experiences in hospitals and social support received (Additional file 1). Data were collected through face-to-face interviews between 45 min and 1 h. The entire conversation were audiotaped and transcribed verbatim in the language of the respondents. Any missing information were checked and updated. Any unclear information was clarified with the respondents.

### Data analysis

The verbatim transcripts were managed using computer aided qualitative data analysis software, namely NVIVO version 10 (QSR International Pty Ltd., 2012). The analysis took place concurrently with the data collection and continued until the saturation point was reached. The respondents were identified by identification numbers to maintain the confidentiality of the findings.

Thematic data analysis, a process of encoding information, was done in six phases [8] as follows. (i) Familiarization with the data: the transcripts were read and re-read to familiarize the researchers with the information and make sure they understood its overall meaning. (ii) Generating initial codes: the entire dataset was coded and labels were generated for important information. The preliminary coding framework was based on the synthesis of initial concepts identified in previous studies. As the process continued, new, emerging codes were anticipated and compared with the initial codes. (iii) Searching for themes: the codes were combined to create subthemes (subcategories) that were then condensed to generate themes (categories). (iv) Reviewing themes: sometimes the themes were split, combined or discarded during the process. (v) Defining and naming themes: meanings were formulated for the categories or themes. (vi) Producing the report: this involved weaving the analytic narrative and the extracts, in which vivid examples were chosen to demonstrate the essence of a point.

Rigour is attained by implementing reliability and validity in its qualitative inquiry [9]. Reliability was attained in this study by reading and re-reading the transcripts to familiarize the researchers and make sure its overall meanings were understood. The transcripts were checked by listening to the audio recordings to make sure errors were not introduced during transcription and coding. During the coding process, data were constantly compared with codes to ensure that no changes occurred to the meaning of the codes. The coding for the same text was cross-checked and agreed to by Zaharah Sulaiman and Asrenee Ab Razak.

Validation is attained in this study by applying triangulation to enhance the research credibility by triangulating various sources. The interview, as the primary data, was supported by the secondary data from home- and hospital-based medical records to ensure the accuracy of information. Any discrepancies between the events or complications reported in medical records and the information obtained during the interview were checked with the respondents. Next, the member checking process was done by phone calls and the polished content of the transcripts and the quotations cited were read to the respondents. Rich, thick description was used to provide many perspectives about a theme so that the results become realistic and richer.

## Results and discussion

Thirty women with maternal near miss events were interviewed between January and December 2014. Table 1 describes the sociodemographic characteristics and Table 2 describes the obstetrics history of the women interviewed. All of the women were Malays between the ages of 22 and 45, of which, 40.0% were 35 years and above. They were married and only one was divorced at the time of the interview. The mean (SD) parity was 2.8 (2.43) ranging from 0 to 10 children. A total of 16.7% women had children more than four. Women experiencing postpartum haemorrhage and heart disease had longer hospital stays. The period of hospitalization was from 3 to 26 days, with a mean (SD) of 8.7 (5.54) days. They were interviewed at approximately 6 days after the termination of pregnancy, just before the hospital discharge, with average duration of 41 min.

**Table 1:** Sociodemographic characteristics (*n* = 30)

Variable	mean (SD^a^)	n (%)
Sociodemographic characteristics		
Age (years)	33.3 (5.60)	
Duration of marriage (years)	8.2 (7.36)	
Household income (RM/monthly)	2982.8 (2475.75)	
Husband age (years)	37.9 (8.24)	
Education level		
Nil and primary		2 (6.7)
Secondary		15 (50.0)
Tertiary		13 (43.3)
Occupation		
Unemployed		11 (36.7)
Self-employed		2 (6.7)
Implementory		10 (33.3)
Professional		7 (23.3)
Husband occupation^b^		
Self-employed		13 (43.3)
Implementory		12 (40.0)
Professional		4 (13.3)
Husband education^b^		
Nil and primary		1 (3.3)
Secondary		19 (63.3)
Tertiary		9 (30.0)
Living with spouse^b^		
Yes		25 (83.3)
No		4 (13.3)

^a^Standard deviation

^b^ *n* = 29

**Table 2:** Obstetrics history (*n* = 30)

ID	Parity	Gestation (weeks)	Condition	Fetal outcome	Mode of termination of pregnancy and surgical procedure
A0025	3	37	Placenta accreta	Alive	emLSCS, Hysterectomy
A0043	2 + 1	34	Placenta increta	Alive	emLSCS, Hysterectomy
A0016	8	37	Placenta accreta	Alive	emLSCS, Hysterectomy
A0046	2	37	Placenta previa	Alive	elLSCS, Hysterectomy
A0097	1	41	Third degree tear	Alive	sVD
A0344	3	39	Uterine inversion, retained placenta	Alive	sVD
A0205	3	40	Retained placenta	Alive	aVD
A0118	4	37	Placenta previa type III	Alive	elLSCS, Hysterectomy
A0248	3	38	Uterine atony	Alive	elLSCS, Hysterectomy
A0210	1	40	Uterine atony, multiple vaginal wall tear	Alive	sVD
A0250	4 + 1	37	Placenta percreta	Alive	emLSCS, Hysterectomy
A0098	1	38	Uterine atony	Alive	emLSCS
A0150	5 + 2	38	Abruptio placenta, hypertensive crisis	Dead	emLSCS
A0221	10	40	Extended tear, broad ligament hematoma	Alive	emLSCS, Hysterectomy
A0134	1	41	Uterine atony	Alive	sVD
A0137	3	40	Abruptio placenta	Dead	emLSCS
A0157	1	41	Multiple vaginal wall tear	Alive	sVD
A0113	5	39	Uterine atony	Alive	emLSCS, Hysterectomy
A0126	8 + 1	40	Injury to parametrium vessels	Alive	emLSCS, Hysterectomy
A0163	3	37	Ruptured posterior wall	Alive	emLSCS, Hysterectomy
A0192	2	35	Severe preeclampsia with cardiac arrest	Alive	emLSCS
A0017	1	29	Eisenmenger’s syndrome	Alive	elLSCS, Bilateral tubal ligation
A0138	1	28	Severe preeclampsia	Alive	emLSCS
A0020	1	34	Severe preeclampsia	Alive	emLSCS
A0119	1	31	Eclampsia	Alive	emLSCS
A0080	1 + 1	11	Ruptured ectopic pregnancy	Dead	Laparotomy, Salphyngectomy
A0193	0 + 1	12	Ruptured ectopic pregnancy	Dead	Laparotomy, Salphyngectomy
A0209	0 + 1	8	Ruptured ectopic pregnancy	Dead	Laparotomy, Salphyngectomy
A0219	2 + 1	12	Ruptured ectopic pregnancy	Dead	Laparotomy, Salphyngectomy
A0260	4 + 1	22	Placenta percreta, intra-abdominal bleeding	Dead	Laparotomy, Hysterectomy

*Note. ID* identification, *emLSCS* emergency lower segment cesarean section, *elLSCS* elective lower segment cesarean section; *sVD* spontenous vaginal delivery, *aVD* assissted vaginal deliver

The findings of the current study shed light on the near death events of Malaysian women following severe and acute obstetric complications. We sought to explore the experiences of women and their perceptions of the quality of care based on the emerging themes. Table 3 summarizes the themes and subthemes that emerged from the data analysis. The four major themes were: (i) self-appraisal of maternal near miss event, (ii) their perception of the quality of care, (iii) their predisposition to seek healthcare and (iv) the social support they received.

**Table 3:** Initial context and subthemes

Initial context	Subthemes	Themes
• Loss of physical function • Loss of blood • Lapse of time	1. Loss of functionality	Self-appraisal of maternal near miss
• Fear • Impending death • Alarm • Anxiety • Incomplete self • Discouragement • Numbness	2. Emotional turmoil	
• Reliance • Content	3. Religious reasoning	
• Child as a motivator to seek treatment • Child as a source of strength	4. Maternal disposition	
• Competency of care • Promptness of care	1. Provision of care	Perceptions of the quality of care
• Interpersonal interactions • Information-sharing	2. Provider-patient communication and relationships	
• Adequacy of staff • Physical infrastructure • Provision of transport	3. Human and physical resources	
• Distance and transportation • Cost	1. Accessibility of healthcare facilities	Predisposition to seek healthcare
• Attitudes • Choices	2. Attitudes and choices regarding healthcare	
• Traditional midwives • Cultural beliefs	3. Traditional and cultural influence	
• Emotional	1. Emotional support	Social support
• Practical	2. Practical support	

### Self-appraisal of maternal near miss

The first theme describes the self-appraisal of the critical childbirth. It is the judgment of the women about the experiences of maternal near miss event. Findings related to self-appraisal fell into four subthemes: loss of functionality, emotional turmoil, religious reasoning and maternal disposition. ***Loss of functionality:*** The women with maternal near miss described their loss of functionality in various ways. Some women talked about their difficulty in breathing, chest pain, lethargy and excessive swelling of their lower limbs (A0119).

One woman, who had experienced paralytic ileus following surgery, talked about her abdominal discomfort and flatulence (A0137). Another woman, who had uterine inversion and retained placenta, talked about the pain when the healthcare provider pushed the uterus back into its place (A0134). One woman experienced the inability to walk, and unlike the other physical limitations, this unexpected, dreadful outcome was thought likely to be permanent.

*“I do not know whether I will recover or not. In the state of being unable to walk... now that I can step on the ground, I feel okay… I did not expect that I would be like this... it’s easy for other people… they can be discharged after three days.”* (A0157)

Many of the women experienced loss of consciousness because of excessive bleeding, seizure or under sedation. They missed the critical events and reported not knowing what had happened during their childbirth. They had no knowledge of what happened to their babies. They woke up in ICUs and realized that surgical procedures had been done, including hysterectomies.

*“When I became conscious, I was in the ICU. The nurses were talking… what date is today… it was the 11* ^*th*^ *… I asked the nurse, are you kidding, it is the 11* ^*th*^ *? I was admitted on the 6* ^*th.*^ *’… The nurse told me that I was not ligated. In fact, my womb had been removed.”* (A0221)

***Emotional turmoil:*** A majority of the women who had experienced maternal near miss reported several forms of negative emotions such as fear, anxiety, alarm, anxiety, incomplete self, discouragement and numbness. Most of the women perceived that death was close. The women adapted to most of these emotions by anchoring their reasoning to religiosity and faith. Generally, the women focused the feeling of fear on their own lives and the lives of their babies. Women expressed fear for the perinatal outcomes, more so in the cases of premature birth. Ten women who had a fatalistic view talked about their fear of undergoing surgery. One woman even feared seeing the nurses and doctors.

*“Thinking there were lots of problem. Death. Then the doctors said that there was a risk of bleeding... have to... might have to remove the bladder... it would be a problem too… the bladder was also injured… if it is a little, then it is alright... but it was a lot… I was afraid I would not have the chance to take care of the children.”* (A0118)

Some women mentioned fear related to the complications of the life-saving procedures. Two women who had undergone salphingectomy for ruptured tubal pregnancies feared of the recurrence of similar incidents and the inability to conceive in the future. One woman feared that the transfused blood might contain the human immunodeficiency virus (A0344) and one woman feared that hysterectomy would cause sexual dissatisfaction to husband (A0221).

Many of the women mentioned the sense of death at the time they experienced the maternal near miss. This feeling was reinforced by the occurrence of bleeding, shortness of breath, cold extremities and pain. Obstetric care, such as emergency caesarean section, laparotomy and admission to the ICU, also influenced the perceptions of the women regarding the severity of their illnesses. With the perception of approaching death, some women asked for forgiveness from relatives and left messages regarding care for the children they would be leaving behind (A0260). Although death was in their thoughts, a few expressed that were not ready to face it yet (A0219). The perception of death was the most striking experience for most of the women with maternal near miss.

One-third of the women expressed feelings of alarm due to the unexpected outcomes of their childbirths. Women described their experiences as traumatic and horrifying when they realized that their uterus had been removed and they were in ICUs following childbirth.

*“I was shocked. It was all gone (referring to her uterus).”* (A0016)

The majority of women posted to the surgical theatre for emergency surgeries reported feeling anxious. Specifically, they were afraid of emergency caesarean sections and being under general anaesthesia. However, those who had been given clear explanations regarding the need for the emergency procedure (A0209) and those who had experienced the same situation in a previous pregnancy did not report this (A0221).

Almost all of the pregnancies had been planned and wanted. A mother without a baby and a woman without a uterus, lead to the feeling of incomplete self. Feelings of loss and unhappiness lingered in these women. They felt as though their babies were still alive, and they would massage their tummies until they were told to accept the facts by their spouse or relatives (A0260). Women who had undergone hysterectomies felt as though they were not complete human beings (A0221). They also reported sadness when they were not able to have any more children. However, all of them adapted by anchoring their beliefs in God’s love and better judgment.

Five of the women narrated that the occurrence of severe, unexpected complications during childbirth discouraged them from becoming pregnant again. This more often occurred among women who had been pregnant with their first child and faced a difficult delivery.

*“Upset with what has happened… others (other women) delivered easily… seven, eight children. If I am strong, I will be pregnant again... if I am not strong, I will accept it as it is… it’s like the end of life… thank God I am alive.”* (A0134)

Another woman who had delivered a second child was also discouraged about becoming pregnant. However, she said she has no choice and will accept it if it is God’s plan to give her more children in the future. Others said they would consider a future pregnancy, provided the spacing between children was a minimum of 2 years to allow for full physical recovery.

Four women were not interested in revisiting what had happened during their traumatic obstetric events with their spouses. These occurred in women who lost their uterus. Due to the severe bleeding, they became unconscious and underwent hysterectomy. They did not feel like discussing the traumatic event with their spouses, whom also advised them to take their minds off it.

*“I did not ask my husband (about the event) because I did not want to remember about it. I am not interested to ask.”* (A0026)

The majority of women who had survived the life-threatening events of obstetric complications presented a set of emotional reactions to maternal near miss. Their fear predominantly related to their survival and that of their children. Fear and anxiety regarding surgical interventions were common and often rooted in a lack of understanding of the need for and the consequences of such procedures. Coupled with the physical loss of functionality, they produced a sensation of imminent death.

***Religious reasoning:*** Women in this study frequently used religious reasoning such as reliance and content as ways to manage or adapt the difficult life events, in this context, the traumatic childbirth. Most of the women turned to religious faith as reasoning and appeared to have accepted the situation calmly. The religious term ‘tawakal’ (reliance) refers to trusting or relying on God. They responded to their situations positively, delegating the resolution to God and regarding what had happened as what God had planned for them.

*“I had to undergo operation (elective caesarean hysterectomy). The uterus was to be removed. Let God help us. We have tried. The rest of the effort is from God. We have to be sincere to rely on God.”* (A0046)

The religious term ‘redha’ (content) refers to wholeheartedly agreeing to a fate God has made. Accepting the situations had brought them to the feelings of relief that the life-threatening events were over and that they had survived the childbirth. They were very grateful that God had given them a second chance (A0113). They felt that God still loved them and as though they had been brought back to life.

*“This is all God’s power. We have to accept. We have to be content.”* (A0017)

The women also took these experiences as an opportunity to correct themselves, place more value on religious practice and build a new life with their husbands and children. Some women viewed their experiences as a test and reminder from God (A0260).

It is interesting to observe how religious faith served as an excellent adaptative process for these women. The women frequently used the religious terms for contentment and reliance on God when describing their reactions to their difficult circumstances. The women conformed to the situation, anchored their beliefs in God and attributed to God their inner growth and the strength to move on with their lives. The women took comfort from their faith or religion and it helped calm them during their health crises [10].

There are associations between religiousness/spirituality and mental health, with greater levels of religiousness/spirituality being associated with better mental health and well-being [11]. However, greater levels of religiousness/spirituality have also been associated with greater levels of negative stress [12]. The latter has been explained thus: women who experienced greater stress during childbirth sought comfort in religion.

Religious coping is widespread and very common among physically ill patients, ranging from 44 to 90%. Religious beliefs have been found to give people a sense of meaning and purpose during difficult circumstances and to promote an optimistic view and provide role models that facilitate the acceptance of suffering. These beliefs offer divine support, which helps to reduce isolation, loneliness and the need for personal control [11]. Other forms of adaptation included natural maternal disposition in which their attention were distracted and focused on their children.

***Maternal disposition:*** Maternal disposition is the instinct nature of mothers that has influenced their behavior. In this context, children can act both as a motivator to seek treatment and as a source of strength. Maternal disposition can be observed when the thought of the baby had changed the decision of a woman whom later decided to seek emergency treatment.

*“She (mother) persuaded me (to go to hospital). I did not want to listen because I wanted to die at home. Oh God, I do not know, only God knows…. She took some time to persuade me. I remembered my second sister; she told me that if I cannot breathe, it will affect the baby. When she mentioned about the baby, I felt touched… I did not want anything to happen to the baby. Then I said, alright let’s go. I went because I was afraid the baby would not get any oxygen.”* (A0260)

Some women focused their attention on their children rather than on their own health, even when facing the risk of death. They focused on their babies as a source of strength to continue living.

*“In that conscious and non-conscious state, I was determined to raise the baby with these hands. There was one feeling, the spirit…”* (A0157)

### Perceptions of the quality of care

The second theme describes the perceptions of the quality of care. It is the perceptions of the women with maternal near miss about the quality of care received at the healthcare facilities. These perceptions fell into three subthemes: provision of care, provider-patient communication and relationships, and human and physical resources. ***Provision of care:*** Provision of care is described in relation to competency of care and promptness of care. Generally, almost all the women were confident and satisfied with the diagnosis and treatment provided by their tertiary healthcare providers. The competency of the healthcare providers in the form of adequate knowledge and skills in providing optimal care had gained trust from the women. The women agreed that their healthcare had been provided by experts. They strongly agreed that the attending doctors in the tertiary hospitals were specialists.

“*Yeah, all of them. They were professors. There were many.”* (A0118)

*“They were the right persons. Those who were more expert, for me. They were the best (doctors) that I have received treatment from.”* (A0080)

Some of the women had less-favourable responses about their antenatal follow-ups in the primary healthcare clinics. One woman recalled a misdiagnosis by the primary healthcare provider. The examining doctor initially told her that she was 5 months pregnant, which the woman, who was multiparous, denied. The subsequent ultrasound finding confirmed a three-month period of amenorrhoea but showed an abnormal growth in the uterus. However, follow-up with a specialist did not reveal any growth. This misdiagnosis could be the results of lack of skill or faulty equipment.

*“The doctor asked why I came at five months because my tummy was big… I told her that if I could get up, that means it’s around three months, four months… she told me to go for a scan; I went for the scan and it’s correct on my side… during the scan, she said that I had a growth… the tummy was big because of the growth… then I went to see the specialist… nope, there’s no growth, said the specialist… the doctor learned on paper… I learned by experience… I felt weird… maybe the computer (scan) was not clear, was spoiled.”* (A0221)

Two of the four women who had histories of ectopic pregnancy expressed similar negative views. These women were dissatisfied with the failure to diagnose their ectopic pregnancies despite frequent visits to several primary healthcare clinics and repeated ultrasound scans. They blamed the private healthcare providers for failing to diagnose their ectopic pregnancies. Delays in making correct diagnoses or recognizing the risk led to delays in obstetric management. Most of these women had been brought to the tertiary hospital in hypovolemic shock and had undergone emergency laparotomy with salphingectomy for a ruptured tubal pregnancy.

Generally, the women were satisfied with the promptness of the treatment they had received upon arriving at the hospitals. Once in the labour room, they had been immediately attended by staff. Only one woman, who had an ectopic pregnancy, felt offended because she was not immediately seen by the healthcare providers in emergency department (A0219).

Almost all the women were satisfied with the promptness of services during their hospitalization. They were impressed with the quick action of the doctors and other healthcare providers in managing emergencies, but one woman voiced worries because staff seemed very young and perhaps not that expert. She thought that senior operating doctors would increase patient confidence (A0138).

The women felt safe to be in the healthcare facilities although the elements of delay were present. One woman, who had arrived with the complaint of severe abdominal pain, said that she experienced profuse bleeding 3 h after admission, while waiting to be transferred to the general ward. Foetal heartbeat could no longer be detected and she was pushed to the operating room for an emergency caesarean section due to abruptio placenta and intrauterine death. The woman felt relieved because she was already in the hospital when this happened and perceived that the actions taken were fast (A0137).

Most of the women experienced long delays in receiving antenatal check-ups in primary healthcare clinics. It took half a day to get this done, with a wait of approximately 2 to 3 h to see the nurses and longer waits to see the doctors. Overcrowding, inefficient staff and facilities under renovation were among the causes. Some women who favoured private clinics stated that the wait times there were much shorter. Antenatal check-ups in primary healthcare clinics were synonymous with long wait times, as expected by many.

*“I went at nine and came back at twelve, one… Feeling like lots of time wasted. Some people advised me to come in the afternoon. There’s no one in the afternoon… “Of course it’s long. It’s the government clinic, right? Moreover, there are many patients.”* (A0097)

***Provider-patient communication and relationships:*** These are described in relation to interpersonal interactions and sharing of information between the women and healthcare providers. Generally, the women had good opinions regarding their healthcare providers. They trusted their knowledge and capabilities and felt secure with the care they received. The responsibility and empathy the providers showed and their willingness to explain the medical problems were perceived as constituting a good quality of care. All the women felt that they were well cared for in the intensive care units (ICU), and this comforted them.

*All the doctors were nearby. They told me that it was an impending death. I’m lucky. I’m lucky. They came one after the other asking regarding my condition. I was monitored continuously... I’m grateful. They cared.”* (A0248)

At the opposite end of the spectrum, some of the women identified the staff as being harsh and judgmental. One woman, who was having her first childbirth, narrated her experience.

*“I felt offended. The doctor asked me, why are you so difficult to deliver? She told me to push. Why is your position like this? Push, clench your teeth, push, push. You were straining at your face, she said. I do not know. I do not know what to do… She asked whether I have any sin with my husband. My husband told her that he had forgiven me everything. Then she said, you are a teacher. What have you done to your students? Why are you so difficult to deliver? Your baby is bigger than your knee… If you do not push, your baby will not get out. At that point, she really made me feel down.”* (A0157)

A single mother, who had a ruptured ectopic pregnancy, described her perception of her healthcare providers. She perceived that the nurses treated her as a secondary priority compared to the other women.

*“If I needed help, they would delay. Yeah, I was not supposed to be in that ward. It was as if I was a problematic girl. They said that I had to swallow it. I had to accept it. Must. I was the guilty one. Their facial expression was like, they did not want, they did not want to look at me... If I were in their place, maybe I would be like that, too… I was like a second priority to them.”* (A0193)

All the women felt that the choices of emergency care decided upon by their healthcare providers were the best options. They appreciated the efforts of their healthcare providers to communicate and share medical information with them or their relatives. However, despite these attempts, seven women did not understand the explanations for the need for such procedures. Some women felt that their understanding was no longer necessary, as they were already in the recovery, but others believed they would feel better if they were able to comprehend the rationale for the procedures and make sense of them.

Most of the women lacked knowledge about the complications of their procedures. They were left with questions and often expected that their healthcare providers should take the opportunity to explain their complications, since the procedures had already been completed. Some of the women wanted to meet the doctors and would have been more satisfied if they could have been seen by the same doctor both before childbirth and while undergoing the procedures for their near miss complications.

Lack of understanding about the recommended emergency treatments instilled fear and misconceptions in some of the women. For example, one woman, who had to undergone removal of her uterus due to uterine atony, was afraid that the non-adherence to the surgical procedure would lead to cancer of the uterus (A0210).

***Human and physical resources:*** These are described in relation to adequacy in the number of staff, physical infrastructure of the healthcare facilities and provision of transport for referring patients to tertiary centres. Most of the women thought that the government healthcare facilities had an adequate number of healthcare providers. The preference for female doctor for delivery was only observed in one case (A0157). The long wait times in the primary healthcare clinics was perceived as being due not to shortages of staff but to the organization of the services and the attitudes of the staff (A0017).

Most of the women regarded the general conditions and equipment at the primary healthcare clinics as highly satisfactory. Only one woman complained of a defective ultrasound scan and haemoglobin-measuring device (A0221). In addition, the hospitals were perceived as being well equipped. However, several of the women had critical opinions and made suggestions for improvement. Some complained that the general wards were congested and hot. Some had brought their own table fans or hoped for air-conditioned wards (A0043). The toilets were limited and situated at the far ends of the wards. Some women feared going to the toilet unattended because of their weakness following childbirth (A0017). One woman wanted adequate parking spaces for her visiting relatives (A0134).

The women were moved from the ICUs to the labour rooms for observation before being sent to the wards. The noise of crying babies, patients and staffs and the small size of the labour-room beds made it less likely for most women to rest comfortably and get adequate sleep. One woman described her discomfort and pain due to frequent changing from bed to trolley or vice versa.

*“Pain, pain, I was already in pain… If I’m not mistaken, I had to change beds three times: once out of the ambulance, then changed again, then pushed into the operation theatre, and then changed again. I was really, really in pain… I could only take deep breaths. That’s all. Take deep breaths.”* (A0080)

The women also mentioned transportation difficulties. Most often, ambulances were readily available and did not have problems travelling long distances. However, two women had experienced delays in reaching hospitals due to transportation problems. In the first case, the ambulance had an accident a few days before and another ambulance had to be called, causing a one-hour delay in transporting a woman at 35 weeks gestation with pre-eclampsia (A0192). In the second case, the delay was related to the ambulance driver. A woman at 37 weeks gestation with pre-eclampsia described her dissatisfaction with the delay in transporting her from the primary healthcare clinic to the tertiary hospital.

*“I was supposed to go at 7.30pm and it was already 9pm. Problems with the driver. The driver was late. When I was admitted (to the labour room), they checked my blood pressure, the doctor called the specialist… my blood pressure was high. Could not wait. Had to do the operation straight away.”* (A0016)

The women in this study were generally grateful for and satisfied with the competency and promptness of the care they received in hospitals. However, the responses regarding medical diagnoses and treatments in primary healthcare facilities were less favourable. These findings should be seen as an opportunity to improve the delivery of healthcare services by enhancing staff training and upgrading the quality of medical equipment. Similar complaints from many patients who visit primary healthcare facilities frequently must be taken seriously and investigated. In addition, private healthcare providers need to improve the care they provide. Diagnosis of pregnancy complications, such as ectopic pregnancies, must be accurate so that women can be referred and managed comprehensively in a timely manner.

Generally, the women in this study reported satisfaction of having reached the hospital for childbirth, and in most of the cases, they were managed promptly and efficiently. The delay that occurred in one case of abruptio placenta resulting in intrauterine death in a tertiary centre was mainly due to the lack of experience and urgency for obstetric emergencies by junior healthcare provider and communication with the superiors. However, they were not dissuaded from seeking antenatal care in government clinics, despite the high number of patients there and the delays in receiving treatment. This is because the services are provided free and the assessments are relatively comprehensive compared to those in private clinics. In the absence of better alternatives to government clinics, the issue of delays remains to be a critical obstacle to the delivery of efficient primary care.

During emergencies, healthcare providers may focus on managing patients and provide less information about the decisions they are making. For the women who experience this, even life-saving procedures generate negative emotions, which may be exaggerated by poor provider-patient communication [13]. In the current study, many of the women spoke well of the attitudes of their healthcare providers, although some reported poor attitudes. Again, these findings suggest that provider-patient communication represents an important element of good medical care that is a part of high-quality medical practice. It is obvious from the narratives that good doctor-patient interpersonal interaction, communication and information-sharing helped the women adapt with maternal near miss events, increased satisfaction and avoided misinterpretations. It is not only the amount of information that matters, but also the type of information. In this study, no gender preference for doctors was obvious, because the women regarded their doctors as expert professionals.

Referral of those with pregnancy complications is a higher priority than referral for admission of pregnancy without complications. Therefore, early arrival at tertiary hospitals for women with obstetric complications is the cornerstone for reducing maternal morbidity and mortality [14]. The current study identified coordination of services and transportation between primary and tertiary centres as practices that often needs to be more efficient.

### Predisposition to seek healthcare

The third theme describes the predisposition to seek healthcare. It is the women and families’ predisposition to seek healthcare during pregnancy, childbirth or postpartum. These predispositions fell into four subthemes: accessibility to healthcare facilities, attitudes toward healthcare, and traditional and cultural influences.

***Accessibility of healthcare facilities:*** Accessibility of healthcare facilities is described in relation to the distance and transportation in obtaining care and the cost of medical care. Most of the women reported that the healthcare facilities were easily accessible with transport and that the journeys from their homes were within five to 30 min to primary healthcare clinics and within 30 to 90 min to hospitals. Antenatal care in government healthcare clinics is provided free for Malaysians, and generally, the women did not have any problems obtaining antenatal check-ups. However, one woman complained about the cost of transportation to obtain her antenatal care. She spent 8.00 Malaysian Ringgit (MYR), or 1.9 United States Dollar (USD), on a public cab for each visit, and occasionally, lack of money would force her to miss an antenatal visit.

*“Sometimes when I did not have money, I did not go (for antenatal check-ups). I have to skip. Have to.”* (A0016)

The majority of the women reported no problems in paying for their medical expenses during their hospital stays. The women who worked as or whose husbands worked as government employees could produce guarantee letters that exempted them from paying their hospital bills. However, other women had to pay in full. Two women reported that they had applied for social welfare assistance to exempt them from the charges or waive the charges and one woman would pay the fees in stages. Some women had saved money for the childbirth, but few were prepared for the additional costs due to their complications. Some expressed their worries about their extended hospital stays, the escalating hospital expenses and the costs for magnetic resonance imaging, blood transfusions, surgeries and intensive care that they and their husbands would need pay.

*“I am stressed now. I want to find the money, but I am in this post-delivery state. Admitted to the ward, operation theatre, and four to five bags of blood transfusions. That is why I urgently wanted to go home. I think it will be expensive. My husband is also stressed.”* (A0205)

In Malaysia, government employees are entitled to free or highly subsidized hospital fees through a guarantee letter that acts as a hospital deposit. Non-government employees are considered full-paying patients and they respond to healthcare costs by mobilizing their financial resources, such as by saving money for childbirth expenses, or by applying for social welfare assistance. The admittance charges or deposits, for medical or surgical cases is 30.00 MYR (7.20 USD) and for gynaecology cases is 15.00 MYR (3.60 USD) [15]. In general, the medical expenses of women with obstetric complications are often higher than the expenses of those without complications. The cost is unpredictable, because it depends on the procedures involved. Although the medical expenses in government facilities are low, this does not imply that all households can afford them. Impoverished households do not always have enough cash to meet medical expenses [16]. The distress of impoverished patients is often evident in their urgent requests to be discharged from the hospital early. Although cost is not within the control of healthcare providers, they can assist by identifying and referring patients for assessment by social welfare agencies.

***Attitudes and choices regarding healthcare:*** This section describes the women and families’ attitudes toward healthcare and their choices regarding healthcare facilities. Almost none of the women had hesitated to obtain care at the hospital. One woman preferred home delivery because she was scared of hospitals, particularly of the needles (A0020). Another woman, even after realizing the seriousness of her condition, delayed seeking help for about 6 h because of her desire to be surrounded by family members and avoid the experience of dying in a hospital (A0260). For certain women, recognizing the seriousness of their condition did not translate into a search for prompt emergency care. These delays are not due to women’s underestimation of the severity of their conditions [17, 18] or to powerlessness to make healthcare decisions [19] but are due largely to the women’s attitudes.

In Malaysia, traditional birth attendants are known as village midwives, or ‘bidan kampung’. In the current study, one woman with an undiagnosed pregnancy delayed treatment and instead sought traditional massage from a village midwife (A0193). All husbands were supportive of their wives accessing healthcare. They accompanied their wives or provided permission, transportation and funds to obtain care. In addition, the women were empowered to make decisions related to their healthcare. In this study, non-certified providers, such as village midwives, rarely play a role in healthcare beyond that of supportive care. Mainly, they provide patients with massage and, to a lesser extent, identify danger signals in pregnancy and help patients obtain care at healthcare facilities. Antenatal health education must increase awareness of the need to seek immediate treatment in the presence of dangerous or abnormal signs.

Despite overcrowding, almost all the women preferred to receive antenatal check-ups in government facilities because of the free and comprehensive services there. Some of the women reported that they had initially gone to a private clinic for confirmation of pregnancy, because it was faster, but had received subsequent follow-ups at government clinics. Most of the women preferred government hospitals for childbirth because of the specialized care and the sense of safety (A0119).

On further probing regarding choice of healthcare facilities and whether finances were an issue, some women said they would have preferred childbirth in a private hospital because of the comforts and personal care available in them. But in cases of emergency, these women reported that they still would have needed to be referred to the government hospitals (A0043).

***Traditional and cultural influence:*** This section describes the role of traditional midwives and cultural beliefs that played among the women during the pregnancy and the postpartum period. Some of the women had sought out village midwives for massage due to their body aches and for the ‘lenggang perut’ ritual. However, in this context, the midwife played a role in advising a woman to seek hospital treatment due to the unresolved pain.

*“I went to the clinic. He told me not to think about it because it was normal. I went home but after that I never felt well. My tummy ached. I was saying it was gastritis. I took medicine and met the midwife. But the midwife did not dare to touch because it was only one month (pregnancy). She only massaged the waist. I felt relieved, but then it ached again. I met the midwife again. Two three times... Finally, I called the midwife and she advised me to go to the hospital…”* (A0219)

Some of the woman believed that consuming pineapple juice and pickles during pregnancy would cause bleeding and abdominal pain (A0193). This was regarded as true by these women because it had been their personal experience (A0260). One hypertensive woman consumed two green apples per day to reduce her blood pressure, but to no avail (A0020).

The majority of the women reported that during their postpartum period they would be on a special diet, would receive traditional massage and ‘bertungku’ (use of heated stone on parts of body). According to custom, women should consume grilled food and avoid fried food, ‘cold’ vegetables such as lady’s fingers, cold drinks, certain fish or chicken. This is done to improve the mother’s general health and to speed the healing of wounds, especially following surgery, but some women practiced this custom for unknown reasons.

*“I have to take care of my diet because I was operated on. I was not allowed to eat chicken, ice… because chicken causes pus. I do not know. I just follow what others say. But once I go back to Johor, I will eat whatever I want.”* (A0209)

There is need is for accurate information during pregnancy and postpartum. Most women adhere to special diets and body massage practices that they perceive will help their recovery during the postpartum period [20]. In one local study, traditional massage was found to protect against postpartum depression; however, prohibition of certain foods has no beneficial effect [20]. Appropriate advice from healthcare providers in general and midwifery nurses in particular can improve patients’ postpartum knowledge, particularly regarding the inaccuracy of food taboos, and can thus accelerate the healing process and an early return to the pre-pregnancy state. Belief in myths can be ameliorated by accurate information.

### Social support

The fourth theme describes the social support. It is the types of social support received by the women during childbirth and peurperium. This social support fell into two subthemes: emotional support and practical support. ***Emotional support:*** The presence of family members such as husbands, mothers or mothers-in-law had provided tremendous emotional support to the women during childbirth in the unfamiliar hospital environments. The majority of the women had shared their feelings with their husbands and some had shared with their mothers and sisters. The majority reported having been satisfied with the emotional support they had received (A0113).

Five women chose not to confide their feelings to anyone and kept them to themselves. Some preferred to express their feelings to God and some chose to ignore their problems. This indicates that the lack of emotional support from close ones was balanced with the religious faith as reported previously. Their reasons included: (i) they felt that others might not understand (A0126), (ii) they did not want others to worry and (iii) they felt embarrassed to express their feelings (A0209).

*“I share it with God... My husband does not understand my feelings... It’s annoying.”* (A0126)

***Practical support:*** While in the hospital, most of the women received assistance from an accompanying person. During the postpartum period at home, most of the women reported that they would be receiving practical help with the household chores and taking care of the baby and their other children. Their husbands and other family members would provide this support (A0097). Only one woman, who is a second wife, would take care of herself, as the rest of the household members would be gone to work or school (A0138).

Social support has been conceptualized in relation to emotional and practical support [21]. Emotional support includes expressions of empathy, trust and caring, while practical support includes tangible aid or services [22]. The maternal near miss events may result in more stress if a woman’s social support is inadequate.

In Malaysia, social support during the postpartum period comes in the form of traditional confinement practice. It is a ritual in which mothers receive mandated rest, a special diet, traditional massage and help with household chores for 40 days postpartum to facilitate recovering their physical and emotional strength. In the current study, no woman identified or described that her postpartum period was going to be difficult. This was not surprising, since the vast majority of the women expected to receive assistance, emotional support and reassurance from close family members and this support was paramount.

Social support and improved relationship quality are associated with better mental health and well-being [11], reduced stress [12] and protection from postpartum depression [23]. In the current study, social support appeared to play a role in protecting the women from ill health. Despite their experiences, the women were relieved at having survived their acute, severe complications and looked forward to resuming their lives normally.

We recommend that apart from providing optimal medical care, healthcare providers must be aware of the importance of adequately informing patients and ensuring that they understand their medical procedures, as this increased the patients’ adaptation process and increases their satisfaction. In addition, acknowledging the benefits of religious faith is important in treatment situations that have potentially fatal outcomes. If a life-threatening complication occurs, full social support (emotional, practical and informational) should be provided and close family should be allowed to accompany the patient and remain with her. The current study showed that these strategies provide psychological support that reduces the suffering of women experiencing maternal near misses.

Malaysia has a well-established programme on Confidential Enquiries into Maternal Death. The addition of a maternal near miss case review programme, Confidential Enquiries into Maternal Morbidity, allows thorough examination of the occurrences, identification of preventable factors and impact on mothers, if addressed, may improve future healthcare and patient outcomes. Confidential Enquiries into Maternal Morbidity, which includes district- and hospital-management teams, appears to be a suitable body to audit care in cases of maternal near miss. Since women who have experienced near misses have survived, enquiries can include case reviews and in-depth interviews. The programme can increase understanding of delays before or during hospitalization, whether due to the woman, to primary or tertiary care factors or to other factors related to providing care or to the predisposition to seek it.

The strengths and limitations of the study are as follow. Respondents were interviewed approximately 6 days after the termination of their pregnancies, therefore minimizing recall bias. For this reason, the researchers are confident that they captured the details of the crucial events. In addition, the self-reported complications were validated against hospital medical records. The findings of this qualitative study have limited generalizability to other populations.

## Conclusion

In appraising the maternal near miss events, the study found that the women viewed their experiences as frightening and that they experienced other negative emotions and a sense of imminent death. Their perceptions of the quality of their care were influenced by the competency and promptness in the provision of care, interpersonal communication, information-sharing and the quality of physical resources. These factors should be of concern to those seeking to improve services at healthcare facilities. The predisposition to seek healthcare was influenced by costs, self-attitude and beliefs.

## Additional file

Additional file 1: Semi-Structured Interview Guide. This is a Semi-Structured Interview Guide used to explore the experiences of women with maternal near miss events and their perceptions of the quality of care. (PDF 7 kb)

## Abbreviations

aVD: Assissted vaginal delivery
elLSCS: Elective lower segment cesarean section
emLSCS: Emergency lower segment cesarean section
ICU: Intensive care units
SD: Standard deviation
sVD: Spontenous vaginal delivery
WHO: World Health Organization
