# Supplementary material for: The experiences of women with maternal near miss and their perception of quality of care in Kelantan, Malaysia: a qualitative study
Source: BMC Pregnancy Childbirth. 2017 Jun 15;17:189. doi: 10.1186/s12884-017-1377-6 (PMC5472946; doi:10.1186/s12884-017-1377-6)
Supplement: Additional file 1: — Semi-Structured Interview Guide. This is a Semi-Structured Interview Guide used to explore the experiences of women with maternal near miss events and their perceptions of the quality of care. (PDF 7 kb) [file 12884_2017_1377_MOESM1_ESM.pdf]

Semi-Structured Interview Guide

**Opening**	Greet respondent.<br>Explain the process of interview.<br>Explain the audio-recording.<br>The respondent will sign an informed consent form.<br>Respondent can refuse or make request to stop the interview at any time.	
**Warming up**	Would you tell me about yourself?	
**Areas**	**Main questions**	**Follow-up questions**
Antenatal Care	Can you tell me about your experience on attending the antenatal care services?	What are the procedures that you have gone through during your visits?<br>How is your understanding regarding the health problems?<br>How about the staff?<br>How long do you wait to be seen by health staff?
Care during childbirth	Can you tell me about your experience on reaching the hospital?	How is the treatment?<br>How is the explanation regarding the procedures?<br>How is your physical ability during the recovery process?<br>How is your feeling?<br>How about the staff?<br>Can you accept what had happened?<br>How is the condition of baby?<br>What is your future fertility desire?
Social support	How is the support from the people around you?	How will be the care during puerperium period?
Other matters	What are your opinions regarding private health facilities?<br>Do you have any suggestions regarding the healthcare service?	How about the medical expenses?
**Closing**	What would like to comment about the interview?<br>Thank the respondent.	
